# An Interview with Hugh Herr

**DOI:** 10.1089/rorep.2023.29005.int

**Published:** 2023-10-19

**Authors:** Marwa ElDiwiny

**Affiliations:** Marwa ElDiwiny, Vrije Universiteit Brussel, Brussels, Belgium. Hugh Herr, Massachusetts Institute of Technology, Cambridge, Massachusetts, USA.

**Figure:**
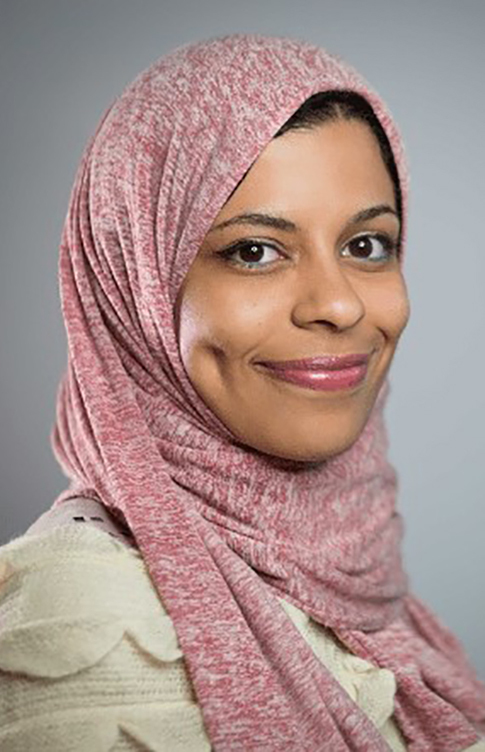
Marwa ElDiwiny

**Marwa ElDiwiny: I would like to go back to when I think you started your career, and it is very inspirational. I am curious about when you started at age 17 years, what kind of rebirth happened to you to start thinking about new bodies?**

**Hugh Herr:** When I was 17 years, I was in a mountain climbing accident and I suffered frostbite to my lower legs. After months of effort, my medical team had to amputate my legs due to the health risk of gangrene. After my legs were amputated, I wanted to return to my chosen sport of mountain climbing, so I designed and fabricated my own artificial limbs for the vertical world. I had specialized limbs for standing on small rock edges, the width of a coin. I had specialized limbs for climbing steep ice walls, and so on. From this body hacking, if you will, I was able to climb at a more advanced level about a year after my legs were amputated than I had achieved before the accident with biological limbs. This was very inspiring, and it led me to my current research objective of human augmentation.

**Marwa ElDiwiny: I admire being creative. I am curious about this motivation and inspiration to design your own artificial limb. How did you start the design?**

**Hugh Herr:** At the time I did not have many resources, and I was not educated in engineering and design, but I was familiar with machining. I spent my high school in a machining curriculum, so I did know my way around a shop. Using passive materials, I fabricated feet, I optimized their geometry, their impedance, their compliance for these various extreme conditions of ice and rock climbing. It worked remarkably well. As I stated, I could climb more difficult faces than I could achieve before the accident. From that experience, I realized that technology has the power to heal, to rehabilitate, and, in my own case, the authority to augment human capability. That inspired me to go back to school and study science and engineering and design.

**Marwa ElDiwiny: How do you define the relationship between the brain and the body? Should the human body necessarily be the same shape all the time?**

**Hugh Herr:** The field of bionics, human bionics seeks to develop synthetic and biological constructs that in the end emulate an anthropomorphic, or normal biological morphology and dynamics. But of course, augmentation is broader than that objective. We can imagine nonanthropomorphic capabilities, humans with third arms for example, or much greater vision, or hearing capacity, or thinking capacity. An example of that is that early on I adjusted the length of my legs, or my stature, well beyond that of a normal stature, to be able to reach handholds and footholds in an accelerated or augmented matter. So even in those early design days, I was exploring this idea of nonanthropomorphic augmentation.

**Figure:**
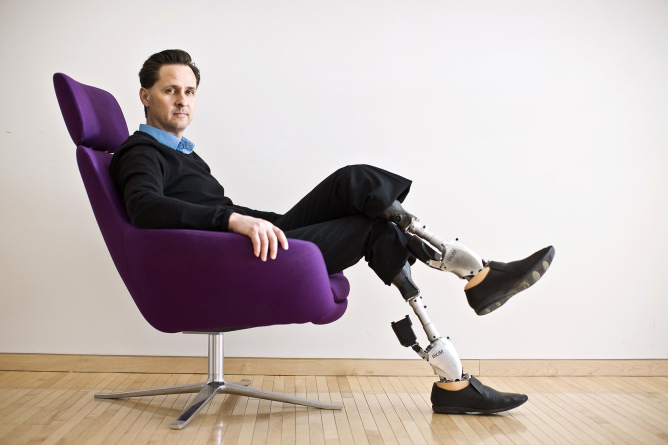
Hugh Herr

As we march into this 21st century, I think that form of augmentation will become more and more paramount. At the twilight years of this century, I predict that humans will be unrecognizable from what we are today, morphologically and dynamically. In my MIT laboratory, we are exploring connecting the human nervous system with synthetic computation bidirectionally. When we do that, we achieve an embodiment, where the human feels embodied into the designed construct as if it is their flesh and bone. That is very interesting, because the design construct does not even have to look like a biological limb or body part. It can be perhaps wings, or any type of form that potentially the nervous system could embody.

**Marwa ElDiwiny: What do you think of this notion of designing something that could surpass what we have already as a human, or Godly creature?**

**Hugh Herr:** Already today we are surrounded by technology that augments human capability. Our smartphones, as an example, airplanes, automobiles, all these devices enable humans to do things they are not able to do with just their bodies. We often do not view it as augmentation, because it is ubiquitous and it is everywhere and we are just used to it. The future happens slowly. We slowly become more and more augmented. Because of that slow speed of augmentation, it is never really surprising. We continually believe that we are not augmented, but we are in fact. My laboratory was the first to develop a leg exoskeleton to augment human walking and running and jumping. That type of technology of augmented humans through exoskeleton bionics we are going to see commercially in just a few years. It is very exciting.

**Marwa ElDiwiny: What struggles have you gone through over the years with the design of prosthetics for different purposes? What is the most challenging part of optimizing the design? To make it adaptable to movement as a human?**

**Hugh Herr:** It is very difficult. Humans are very proficient at walking. We have been doing it for a very long time. The idea of developing a machine that makes us even better at walking and running and jumping is an extraordinary task. Our primary strategy is to try to determine where humans are inefficient and where we are efficient, and where we are inefficient, using technologies in a clever way to augment, to improve the efficiency. For example, when muscles and skeletal muscles do positive thrusting work, they are very inefficient, the muscle gives off a lot of heat.

We look at walking and running gates. We look for areas of the gate for which muscles are doing a lot of inefficient work, and then we replace that work with a motorized exoskeleton capability, to effectively reduce the amount of food energy or metabolics used to move. Imagine a leg exoskeleton where one could run through the wilderness across rough terrain without even breathing hard, and not being particularly in shape. When that is invented, no one will ever use a wheeled mountain bike ever again. If we capture both the versatility of human legs and we improve the efficiency or economy of movement through exoskeletons, it will be a remarkably fun toy.

**Marwa ElDiwiny: What is missing to achieve this?**

**Hugh Herr:** To solve bionics, a few challenges remain. One is the mechanical interface between the bionic synthetic device and the human body. How do you attach a device to the human skeleton, or body, in a comfortable manner? A second interface is neural, how do we connect the brain to bionic devices? And the third, I suppose, is the capacity to build bionic devices that move like us, which relates to power supplies, and muscle-like actuators, if you will. So those three interfaces, the last being dynamic, the second I mentioned being neural or electrical, and the first being mechanical, are the key challenges of physical augmentation bionics.

**Marwa ElDiwiny: What is still challenging to understand about locomotion? How can you make this connection between biology and bionics?**

**Hugh Herr:** To be cyborg suggests a bidirectional connection between artificial computation and the biological brain. In the case of a prosthesis, the human can think and have affect, put information into the synthetic computer, and in turn the synthetic computer can insert energy back into the nervous system. Closing the loop creates the cyborg function. What are the challenges? There are many challenges, but the dominant one we are working on is how to create that bidirectional link between small computers and an interface to muscles and nerves, in terms of information signaling. We are looking at ways of connecting to nerves and muscles, novel surgical strategies, for example, novel implants into the periphery to record these various signals.

**Marwa ElDiwiny: Why choose the bionic option over the cyborg?**

**Hugh Herr:** Bionics is perhaps a subset of cyborg functions. Cyborg simply means that the brain is connected to the mechatronic device bidirectionally. Cyborg does not necessarily suggest that it is biological-like, or human-like, which bionic does, human bionics does. You could be bionic but not a cyborg, and you can be a cyborg but not bionic. There is distinction in my view.

**Marwa ElDiwiny: Don't you think there is an advantage for using cyborgs now?**

**Hugh Herr:** Yes, the bidirectional link to the brain, there are profound advantages. Our brains are remarkably adaptable. It is remarkable what we can do from a motor physical perspective. From an exoskeleton prosthetic perspective, that cyborg bidirectionality is critical for embodiment. If you want to give a person, for example, with limb amputation, the feeling that the synthetic limb is actually themselves, is part of their body, then I believe strongly that the bidirectional brain linkage is central.

We in the field could continue to advance robotics and AI, and build purely intrinsic prosthetic limbs that are highly adaptive and emulate very closely biological dynamics. The problem with that approach is that the human would never feel connected to the device and would never feel embodied. It would feel like, as an analogy, as if it were always sitting in the backseat of the vehicle, but you would never be the vehicle, or with your hands on the steering wheel. It is very important, as a field, that we pursue a high-fidelity linkage between the human brain and synthetic computation elements that sit on the bionic structures, because only through that will we seek embodiment, where persons with limb loss after going through bionic reconstruction will truly feel that they have their body back. That is the clinical goal.

**Marwa ElDiwiny: Do you think the approach should be invasive or noninvasive? Which one do you think is more reliable to the way of the communication between the brain, if we speak about bidirectional communication here?**

**Hugh Herr:** If we consider the spectrum of noninvasive to highly invasive, there is an intermediate stage of minimally invasive. The question is, across that spectrum, what are the advantages and disadvantages? Typically, purely noninvasive interfaces have a very poor signal quality, and they can also be uncomfortable. There is a limit in comfort. There is a limit to how much you can extract from the signals that are collected with noninvasive electrodes, for example.

The advantage of highly invasive is you get beautiful time and variant signals, but it is invasive and may require surgeries, and tremendous cost and whatnot. The minimally invasive point, intermediate point, is very interesting, because it requires minimal surgery, but you still can get sufficiently good signals in terms of quality. In my laboratory, we are exploring minimally invasive neural interfaces to muscle, for example. Our approach is to implant small spherical magnetic beads into muscle. Then we are able to, with external electronics outside the body, measure how the muscle moves dynamically with very high accuracy and precision.

**Marwa ElDiwiny: Do you think the material part of this connection feels like part of your body, and it does not matter whether it is a biological part or a synthetic part?**

**Hugh Herr:** If we solve the linkage between the human nervous system and mechatronics, from the perspective of the human, it will not matter that their limb may be synthetic. If you completely maintain the signals coming out of the brain and into the brain, the central brain will not know what the limb is made of. We are already seeing this in our work.

In a sense, if you connect to the nervous system well enough, the brain will not even realize that the limb has been amputated and rebuilt with synthetic materials. The assumption there is that we have completely maintained the information flow into the brain, and that requires fantastic sensing in the artificial construct that is biomimetic, and it requires a high bit rate information flow into the nervous system. These are all tremendous challenges, and the material and the sensor design is very important for that goal.

**Marwa ElDiwiny: When you try to upgrade each version, what are you looking for in the performance?**

**Hugh Herr:** My laboratory is very diverse from an intellectual and skillset perspective. We are approaching the problem from most directions. It is perhaps one of the most diverse laboratories in the world, in the area of bionics. We are doing genetics, we are doing machine learning, motor design, power supply design, and tissue engineering. Across the board, we are integrating these formal disciplines, and creating these bionic platforms.

The human body is very diverse, the human body is chemical, it is mechanical, it is electrical, and so on. To hack the human body, intellectually, the team has to be very diverse or that team will not be successful. In addition, beyond science and technology, the team also needs to comprise designers and artists. If you are reimagining the human form, we want to also think about aesthetics, and not just functionality.

**Marwa ElDiwiny: Was there something through the years that was very challenging to design, and maybe was not intuitive, and maybe ended with something very beautiful in design?**

**Hugh Herr:** With artificial limb design, what we typically do is we design a limb that has the shape of a normal biological limb, but when you peel off the artificial skin, it does not look biological at all. We do that because the human user of that limb may one day want to hide the fact that part of their body is artificial, and will use that synthetic skin. The very next day, they may have a different mood and want to go to an interesting party and celebrate the fact that part of their body is a machine, and exhibit a machine-like beauty. Allowing the user to explore both a human beauty and a machine beauty from day to day is what we would like to see in terms of our design goals.

**Marwa ElDiwiny: When you consider the intelligence and the design, and you think of something like reflexes, for example, how do you adapt to uncertainty, to situations that may not be expected?**

**Hugh Herr:** The challenge of a purely intrinsic design, where all the sensing and computation is on the synthetic device, the challenge of the unexpected banana peel on the floor is very difficult. If you link the brain bidirectionally to the synthetic limb, you essentially solve that problem, to the degree to which the biological system is capable of adapting, capable of tremendous versatility. You tap into that intelligence, and, if you will, get it for free. That is another reason why, I think as a field, we first need to solve the interface with the brain.

Once we do that, we can use artificial intelligence to augment humans beyond natural capabilities. But first and foremost, we need a high-fidelity linkage to the brain. The dominant challenge is the link between synthetics and biologics. If you want to collect signals from a nerve, motor signals to extract how the person wishes to move, and if you want to reflect the sensory information from the bionic appendage onto the nervous system, the actual connection to the nervous tissues is the challenge. There has been tremendous progress in the past decade in that regard.

**Marwa ElDiwiny: What type of creativity in the design do you envision for what is next?**

**Hugh Herr:** Our approach is called neural embodied design. Most designers and bionics, they think about the design of a synthetic device that somehow interfaces inside the body or is attached to the body. They do not think about fundamentally redesigning the body. With neural embodied design, we not only design synthetics, but we also design the biological body. We explore that interplay between biological design and synthetic design to try to maximize the bidirectional communication between the brain and the device. Because of that, we invent surgeries, we invent new proteins to be able to turn on and off cells. All of these areas where we do not view the body as invariant, we view the body as an area for which one can design. Because of that philosophical distance, I believe we are seeing tremendous success in neural interfacing.

**Marwa ElDiwiny: Where do you think maybe the advancement should lie in the next generation for exoskeleton or prosthetics?**

**Hugh Herr:** My definition of “exoskeleton” is a device that typically runs in parallel to a limb, your leg, for example, that comprises motors and structures to apply in impedances and positions and torques on the body. An exoskeleton is typically used for human augmentation, whereas an orthosis is used for medical purposes, for rehabilitation.

Many of the same challenges exist between exoskeletons and medical prosthesis. Once again there is the goal of how do you extract the intent of the human, how the human wishes to move? How do you insert information back into the nervous system to give the human brain information about the state of the mechatronics and the biological body? How do you attach to the body mechanically? Building an exoskeleton that touches us from the outside, and exerts forces and torques, is very hard, because humans are very soft. You are pushing on soft tissue, and it is very hard to efficiently apply torques about the body with such a soft substrate.

These are all tremendous challenges. As I mentioned, my laboratory was the first to build a leg exoskeleton that augments human walking and running. Believe it or not, that occurred in 2014. For more than a century, scientists and technologists tried to augment humans with exoskeletons, and experienced failure after failure. It was not until 2014 that there was a success. As I stated earlier, humans are very good at walking, we are very good at moving. To build a machine to that makes us even more efficient, or more economical, is a tremendous feat.

**Marwa ElDiwiny: Did you have any surprising or unexpected moments?**

**Hugh Herr:** As a researcher most days are very frustrating, but there are some days that are magical. My group invented a new way of amputating limbs, called the Agonist-Antagonist Myoneural Interface, or AMI for short. In the surgery, we dynamically link muscles to create a biological joint within the amputated residuum. We link sensors to those muscle, dynamic muscle pairs to control the bionic limb.

**Figure:**
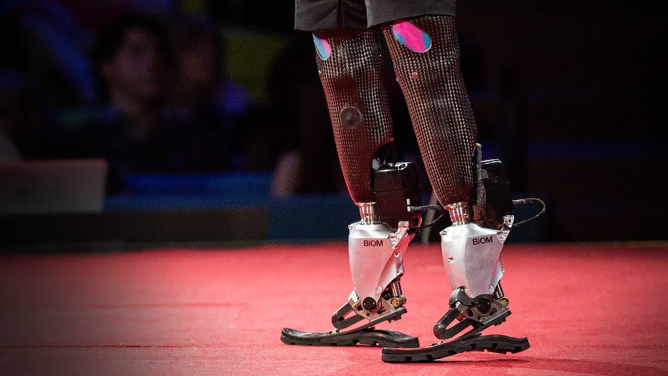


Our first human patient, when we wired him up after his surgery, was quickly able to control the bionic ankle, and bionic subtalar joint. He was moving his bionic foot–ankle complex. We were excited about that, because that in itself was novel. Then he stood up, and he began traversing slopes, and steps, and various irregular surfaces.

His synthetic limb was adapting to the terrain as if his limbs were biological. When we asked him, “Are you trying to move your limb in that way?” he answered, “No, it's involuntary. I'm not conscious of the movements at all.” That was an extraordinary day in the laboratory, because we realized that if you give the brain natural proprioceptive signals, the human brain knows exactly how to control the synthetic limb as if it is a natural biological limb, without even the human being aware that the movements are happening.

**Marwa ElDiwiny: Sometimes with design, there is a trade-off. Is there some limitation or trade-off in the design?**

**Hugh Herr:** In my laboratory, the goal was to not only expand human knowledge, but also to ultimately develop technologies that humans can use to translate ideas into clinically viable technologies. That is a huge challenge to innovate. That design matrix includes not only elements of functionality of the device or system, but also cost and manufacturability. Whether the device can be reimbursed, whether the device has safety that can get through the FDA, for example. The complexity of building a bionic body part and translating it to society is enormous. It typically takes me a decade to build, design, and launch a bionic body part. Whether it is a foot, or a knee, or a hip, or whatever, typically it takes me a decade to innovate. That is how hard it is. There are many trade-offs that one needs to make in that extraordinarily complex effort.

I mentioned earlier that we have highly invasive interfaces to the brain, and interfaces that are completely noninvasive, and then in between is minimally invasive. Minimally invasive is a beautiful trade-off because you have minimized the level of invasiveness. Yes, it requires a surgery, but it is on an outpatient basis. The regulatory hurdle is modest, the cost is modest, but still the signals have very high quality. We do think of these sweet spots in terms of trade-offs, where we can have high performance, and we can also translate the technology to society in a very efficient manner.

**Marwa ElDiwiny: What other things do you wish you could design or engineer, but you maybe do not have the capacity to do so yet?**

**Hugh Herr:** Very few things are impossible. The only futures that are impossible are those futures that are counter to physical law. I spent decades trying to translate technologies, trying to innovate and failing, and only fairly recently have I succeeded. It takes a comprehensive skillset to be able to translate technology, and it takes many people. It takes many creative individuals working together and working very hard for it to be possible. A group of people with a shared vision is essential, with sufficient capital to make it happen.

**Marwa ElDiwiny: What is your secret to having the perseverance to make something happen?**

**Hugh Herr:** If you study highly creative, highly innovative people and institutions, one discovers that they have certain characteristics. Being a force of nature is one characteristic, which means never ever giving up, believing in your vision so acutely that you just know it is possible, and refusing to stop until it is solved. Another characteristic is people who are highly innovative and creative, they do not have the language of failure, they have the language of exploration. If I build a bionic foot, and I test it and it fails miserably, to me that is not failure, it is exploration.

When something breaks or does not perform as one expects, you are that much closer to solving the puzzle and achieving that high performance that you seek. When I build a bionic limb and it performs in a way that is not consistent with the dream, I jump up and down with excitement. Whereas someone else may say, “Oh, I failed. I guess I'll give up.” To me when it does not perform, I am that much closer to success. it is exciting, it is an exploration, it is not failure.

**Marwa ElDiwiny: Do you expect regenerative biology to be a competitor for prosthetic technology in the long run?**

**Hugh Herr:** A friend of mine is a synthetic biologist and he says, “Hugh, why are you wasting time trying to build limbs out of synthetics? Why don't we figure out how to grow them back?” That is an old-fashioned view, because very soon, for example, our synthetic artificial muscles will be far superior to biological muscle in terms of force generation and energy usage and whatnot. I do not view that the biological limb in its innate form is the best we can do. I think technology will become extraordinary, and, therefore, we should continue to think about this merging between electromechanics and tissues, because that ultimately in 50 years will result in the highest performance.

**Marwa ElDiwiny: Why cannot we have a tentacle-like leg, which can be flexible-ending and adaptable to different environments?**

**Hugh Herr:** There are all kinds of novel sensing strategies that one can envision, and that are being pursued in laboratories across the world And a lot of the sensing, artificial sensing has biological counterparts, little hair cells if you will, that are synthetic, that somewhat work like biological hair cells, would be an example. Bionics is a wonderful field to work in, because nature has provided us such a vast array of capabilities, just glorious and beautiful capabilities, and it is tremendous fun to try to use engineering design to emulate those capabilities.

**Marwa ElDiwiny: I have a huge respect for researchers in academia that are trying to make a positive change in people's lives. Do you see the overall research in academia and funding, especially in robotics, going in the right direction?**

**Hugh Herr:** To not only expand human knowledge, but also to build devices that can translate to society, is very challenging. The key to success is diversity. You need an extraordinary intellectual diversity represented in your team. The human body encompasses so many domains of chemical, mechanical, electrical, and so on. You also need designers and artists. You need business minds as well, and you need a lot of capital. The problem with some areas of academia is the laboratory perhaps does not have that diversity.

Then nothing from the laboratory is ever translated to society, because inevitably a mistake will be made in the design, and it will not be a device that can be commercialized. Whether the mistake is a device that is too costly, or does not fit, or does not perform properly, mistakes are often made when there is a limit on the diversity and the scope of intelligence of the full team.

**Marwa ElDiwiny: If someone is interested in studying biomechatronics, do you think he or she needs to have a huge depth of knowledge in physiology? What does it take to be efficient and understand the problem?**

**Hugh Herr:** Some laboratories are filled with incredible engineers, and some laboratories are filled with remarkable scientists of biology, and neither of those types of laboratories tend to be terribly successful. The scientists do not have the engineering sophistication, and the laboratories with engineers do not have the scientific sophistication. The key is to have sophistication in both science and technology, purely from a device functionality perspective. If you also want to translate, you also need experts in regulatory, clinical work, reimbursement, formation of companies, and so on.

Often projects fail because an insufficient level of expertise exists at the beginning of the project, and then mistakes are made, and the project just ends. The key is to have many voices, many perspectives sitting around the table at the very beginning of the initiative.

**Marwa ElDiwiny: I am curious to ask what makes you fulfilled in that what you are doing? What fuels your passion to keep going?**

**Hugh Herr:** It is very powerful that I use prostheses myself. That is important on a few levels. One is that I am intimately aware of the consequence of poor design. I have spent my life trying to work with bad design, and I know firsthand how devastating and painful it is to be trying to go about the world and you are surrounded by technology that is incredibly poor in its conception and design.

Another aspect is that I am often the first human to test the technologies that are being conceived in my laboratory. I am the first person to feel it, to experience it. Because I can feel it and I also know the physics and engineering in my head, I can quickly find the bug and solve the bug. Whereas another person may know the science and the engineering, they cannot feel it. So they do not come to the solution as quickly as I do. That is an interesting advantage that I have.

Regarding my passion, again, I know how painful it is to be in a world with design that does not work. I know how extraordinary it is, and life-changing it is to be given the gift of a fantastic design, whether it be a beautiful chair, or a prosthesis, or exoskeleton. Great design leads to just absolute joy in human expression. What really drives me and my passions and whatnot is the times in my life where I can put out into the world a fantastic design that improves people's lives.

**Marwa ElDiwiny: What kind of future do you think about?**

**Hugh Herr:** In the short term, I would love to see a person with leg amputation dance ballet. I would love to see a person with no arms play a Beethoven sonata, at normal speeds, with normal expressions. Longer term, I would like to see a world where society does not have such a narrow view of human intelligence and human beauty. A world in which we greatly expand human diversity, in which our notion of what is beautiful and what is intelligent greatly expands.

Now, society has a very narrow view of what a beautiful woman looks like, and a beautiful man looks like. We have a very narrow view of what an intelligent person is. Imagine a world, where I hope we will be in 50 to 100 years from now, where humans can express all kinds of shapes and morphologies and dynamics, all forms of thinking, and expressing, and feeling. As we blend our physiologies in with the built environment, we will have this greater capacity to express. My hope is that we will expand human diversity, not shrink human diversity, and fundamentally change our view of beauty and expression.

**Marwa ElDiwiny: I am curious about what life means to you. Is there something you would like to change, or in retrospect when you look, “That's something I would like, if I have opportunity, I would like to?”**

**Hugh Herr:** I believe that we can, in this 21st century, solve disability. Now, because of poor design, humans across the world experience disability. My hope is at the twilight years of the century, that our technology is so sophisticated that humans no longer experience disability. It is a human rights issue as I see it.

Today, we live in a world where if you are not able to see, you are not able. There is insufficient technology to allow you to see. If you are not able to hear the way you wish to hear, there is insufficient technology to allow that. If you are paralyzed and you want to dance, there is insufficient technology to allow that. In the future, I hope we all have the human rights through advancements in technology to be able to see, to hear, and to move, and to live a life without severe depression, for example, if we so choose. Today, that is hard to imagine, because we are so used to a humanity that simply needs to live with profound experiences of disability and limitation. It is hard to even imagine a society without that. But that is my dream.

**Marwa ElDiwiny: Wonderful, I have huge respect for you. Do you have any final words you would like say to the robotics community?**

**Hugh Herr:** Sometimes we make the mistake of saying, “There's this certain cohort of people that are creative, and the rest of humanity, well, sorry, they just weren't born with the right genetics.” I do not believe that. I think creativity is an emotional way of being that is largely cultural. That is tremendously good news, because as parents, we can teach our children, and as community leaders, we can teach our communities, to be more creative and expressive, and to be part of the global effort to solve tremendous problems. Whether it be in disability, or the environment, or politics. I will leave you with that thought.

**Marwa ElDiwiny: Thank you so much.**

## Funding Information

Ms ElDiwiny is supported by the EU Marie Curie ITN project SMART (860108).

